# Online Acceptance and Commitment Therapy for People with Painful Diabetic Neuropathy in the United Kingdom: A Single-Arm Feasibility Trial

**DOI:** 10.1093/pm/pnaa110

**Published:** 2020-05-01

**Authors:** Kitty Kioskli, Whitney Scott, Kirsty Winkley, Emma Godfrey, Lance M McCracken

**Affiliations:** p1 Health Psychology Section, Psychology Department, Institute of Psychiatry Psychology and Neuroscience, King’s College London, London, UK; p2 Guy’s and St Thomas’ NHS Foundation Trust, London, UK; p3 King’s College London, Florence Nightingale Faculty of Nursing, Midwifery & Palliative Care, London, UK; p4 Uppsala University, Psychology Department, Uppsala, Sweden

**Keywords:** Painful Diabetic Neuropathy, Acceptance and Commitment Therapy, Feasibility Trial

## Abstract

**Objective:**

This study aimed to assess the feasibility of online Acceptance and Commitment Therapy for painful diabetic neuropathy in the United Kingdom and to determine if a larger randomized controlled trial testing treatment efficacy is justified.

**Methods:**

Participants with painful diabetic neuropathy were recruited online and from hospital services. This was a single-arm study in which all participants received online Acceptance and Commitment Therapy. Participants completed questionnaires at baseline and three months post-treatment. Primary feasibility outcomes were recruitment, retention, and treatment completion rates. Secondary outcomes were pre- to post-treatment effects on pain outcomes and psychological flexibility.

**Results:**

Of 225 potentially eligible participants, 30 took part in this study. Regarding primary feasibility outcomes, the treatment completion and follow-up questionnaire completion rates were 40% and 100%, respectively. Generally, at baseline those who completed the treatment, compared with those who did not, had better daily functioning and higher psychological flexibility. With respect to secondary outcomes, results from the completers group showed clinically meaningful effects at post-treatment for 100% of participants for pain intensity and pain distress, 66.7% for depressive symptoms, 58.3% for functional impairment, 41.7% for cognitive fusion, 66.7% for committed action, 58.3% for self-as-context, and 41.7% for pain acceptance.

**Conclusions:**

This preliminary trial suggests feasibility of recruitment and follow-up questionnaire completion rates, supporting planning for a larger randomized controlled trial. However, treatment completion rates did not achieve the prespecified feasibility target. Changes to the treatment content and delivery may enhance the feasibility of online Acceptance and Commitment Therapy for people with painful diabetic neuropathy on a larger scale.

## Introduction

Painful diabetic neuropathy (PDN) is a complex pain condition associated with diabetes. It affects ∼25–30% of people with diabetes [1,2]. The main symptoms are tingling and burning sensations in the hands and feet that can have a significant impact on daily functioning [2,3]. Psychosocial factors, such as depression, anxiety, and sleep are significantly associated with PDN [3]. At the same time, current treatment options are mainly pharmacological and appear to produce limited benefits [4]. The experience of pain, and how pain is viewed by others, may differ in this population compared with other populations suffering from chronic pain of mainly musculoskeletal origin [3,5].

Acceptance and Commitment Therapy (ACT) is a newer contextual form of Cognitive Behavioral Therapy (CBT) that incorporates acceptance, mindfulness, and values-based behavior change [6]. It specifically focuses on increasing psychological flexibility (PF) [7], which includes six processes: acceptance, cognitive defusion, awareness of the present moment, self-as-context, committed action, and values-based actions [8].

Systematic reviews show that CBT is effective for chronic pain in general [9]. ACT has a growing evidence base for the treatment of chronic pain and appears to produce outcomes similar to traditional CBT [10,11]. ACT appears to produce better results post-treatment regarding pain-related disability in comparison to alternative treatments, such as relaxation [12].

ACT has not previously been evaluated in PDN [13]. It is designed to be broadly applicable to different types of psychological and physical problems and may be particularly suited to multiproblem cases. Therefore, ACT may be a good fit to address the multiple impacts of pain and the range of physical and psychosocial comorbidities that people with PDN can experience [13,14]. Additionally, ACT assumes that targeting a core set of behavioral processes (i.e., PF) can lead to improved functioning and quality of life generally across these different problem areas. Thus, ACT for chronic pain may also help people with PDN without requiring specific adaptations.

A current challenge is that access to CBT and ACT for pain management is limited outside of specialist centers [15]. However, online treatments may address this, and they may be cost-effective, time-efficient, more acceptable, and less stigmatizing than face-to-face treatments [16,17]. Several studies have investigated online CBT and ACT for chronic pain, all yielding moderate to large improvements in pain and disability compared with waitlist controls or other psychological treatments [15–20].

No studies have examined online ACT for PDN, despite the clear need, potential to enhance access, and potential for cost-effectiveness. Therefore, the current study aimed to test the feasibility of online ACT for people with PDN within the context of a single-arm trial to identify if a larger randomized controlled trial (RCT) would be possible and justified. The feasibility questions were whether online ACT would be acceptable to the PDN population, as reflected by adequate recruitment, follow-up questionnaire completion, and treatment completion rates. For each of these questions, a priori criteria were set against which to determine feasibility. In terms of secondary feasibility questions, effect sizes were calculated to determine whether participants who received ACT treatment would improve on pain outcomes and PF.

## Methods

### Trial Design

This study was an online single-arm (nonrandomized) feasibility trial. The treatment being tested was originally designed for individuals with chronic pain in general. NHS ethical approval was obtained from the Surrey Research Ethics Committee (29/1/2018, Ref: 17/LO/2047). All participants gave informed consent, and the protocol was registered at clinicaltrials.gov (NCT03700528). The study followed the ethical standards of the Declaration of Helsinki (1964) and its later amendments.

Participants completed assessment at baseline and three-month follow-up through a secure survey platform (Bristol Online Survey [BOS]). Even though the literature recommends RCT designs [17], the National Institute for Health Research [21] highlights that not all feasibility trials should be randomized. Our focus was on recruitment, retention for follow-up questionnaires, and treatment completion rates, which are aims that do not necessarily require randomization.

The total sample size was calculated to allow reliable estimation of retention and completion rates, assuming a retention rate of 80%. The estimated sample size would allow for estimation of the true population consent rate with an 11% margin of error (95% confidence interval) for eligible participants. Past research in chronic pain conducted by the team suggests consent rates between 50% and 70%, assuming a more conservative uptake of 40%, and that ∼30% will meet the eligibility criteria. Additionally, a sample of 30 participants is in line with recommendations for feasibility trials [22].

### Recruitment and Participants

**Figure 1:**
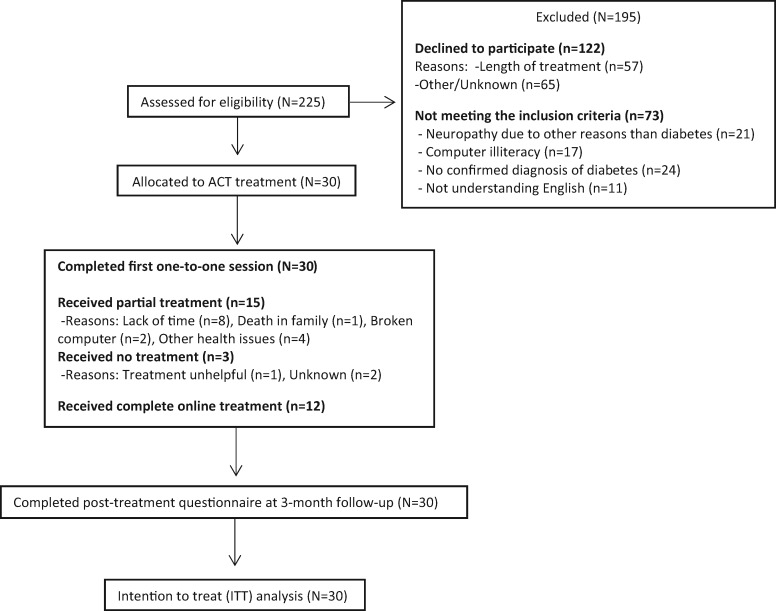
Study flow diagram.

The case definition was adults with PDN. The main inclusion criteria were 1) ≥18 years old; 2) diabetes and PDN diagnoses, which were identified through self-report questions, the Douleur Neuropathique 4 interview (DN4i), and a physician’s diagnosis, when available; 3) verbal and written proficiency in English; and 4) computer literacy. Potential participants were excluded if their primary pain was not PDN. Please see Figure 1 for recruitment details. Participants were recruited via Guy’s and St Thomas NHS Foundation Trust and online advertisements. Online invitations were sent and resulted in recruitment as follows: “Diabetes UK” (https://www.diabetes.org.uk/research/take-part-in-research; N = 15), “Pain Support” forum (https://painsupport.co.uk/; N = 8), “Pain Concern” forum (http://painconcern.org.uk/how-we-help/forum/; N = 4), and Twitter (N = 1). Final post-treatment questionnaires were collected in April 2019.

### ACT Online Treatment

The purpose of this online therapist-supported treatment was to increase participants’ PF—namely, their willingness to experience pain, awareness of experiences in the present moment, and engagement in committed and values-based actions [10]. ACT was considered appropriate for this study, as PF is a transdiagnostic model and can be applied to various conditions with no need for any alterations [23]. ACT is theoretically well suited to a range of problem areas, and on average people with a range of conditions benefit.

Online treatment procedures and content were based on the online version of ACT, developed and initially tested by Scott et al. (2018) [15]. The online treatment platform that was used was called ACT4PAIN, initially created by LM and WS. The treatment involved one 30–45-minute Skype session with the therapist at the beginning of treatment to explain the treatment processes and set therapeutic goals. In the current study, the first author (KK) acted as the therapist. The therapist’s experience level was Master’s-level in health psychology, six months of certified training on third-wave CBT from the British Psychological Society (BPS), National Health Service (NHS) training on Good Clinical Practice (GCP), and further training from LMM and WS, who are registered clinical psychologists with experience providing ACT for chronic pain. WS provided ongoing supervision to discuss participants’ engagement and challenging responses as they arose. As KK acted as the therapist and analyzed the collected data, direct data entry from each participant and remote/online assessment were used to reduce the influence of the researcher on the assessment.

Following the first Skype session, eight online sessions were provided in a five-week period. This standardized package was delivered, two times per week for the first three weeks and one time per week for the final two weeks. The delivery was conducted according to the originally developed treatment by Scott et al. Twice-weekly sessions were chosen earlier in the treatment to keep participants focused on the treatment and practicing new skills. This was based loosely on a previously designed treatment [24]. Frequency of treatment sessions tapered off in the final two weeks to foster greater independence in preparation for self-management after treatment completion. The sessions consisted of video- and audio-recordings that guided participants through experiential exercises, mindfulness practice, metaphors, values clarification, and values-based goal setting. Online sessions included video and audio content that was between 12 and 35 minutes in duration (see Tables 1 and 2 for more details on treatment content). The total approximate time for the content delivered from the system was ∼150 minutes.

**Table 1:** Summary of Acceptance and Commitment Therapy psychological treatment sessions

Sessions	Information	Tasks/Exercises	Total Video Running Time	Total Audio Running Time
Session 1: Skype one-to-one	Introducing the treatment	Goal setting & Identify barriers	–	–
Session 2: Online	Living with pain: Shifting your focus	Passengers on the bus metaphor & Notice 5 things exercise	6.16 min	10.86 min
Session 3: Online	Open: Letting go of the struggle with pain	Unwanted party guest & Connect, breathe, open up exercise	3.99 min	12.16 min
Session 4: Online	Open: Responding differently to thoughts	Mind experiments & Labeling thoughts exercise	7.01 min	17.63 min
Session 5: Online	Engaged: Choosing your values and goals	Choosing your focus & 80th birthday exercises & Values assessment form	6.32 min	10.52 min
Session 6: Online	Aware: Focusing on the present moment	Tracking thoughts in time	7.23 min	27.08 min
Session 7: Online	Engaged: Committing to your goals	The swamp metaphor, Small steps exercises, & Goal-setting form	3.84 min	8.06 min
Session 8: Online	Aware: A different point of view	“Observer self” exercise	4.94 min	17.32 min
Session 9: Online	Building wider patterns of success	“Brief observer self-exercise,” Your kind friend exercise, & Goal-setting form	4.16 min	10.87 min
Session 10: Skype one-to-one	Committed action & Debriefing	Goal setting & Evaluation	–	–

This format is based on the treatment in Scott et al.’s trial [15].

**Table 2:** Overview of treatment’s schedule

Week (0)	Week (1)	Week (2)	Week (3)	Week (4)	Week (5)	Week (6)	Week (7)	Week (12)
Participants give informed consent and respond to the baseline questionnaire	Skype session with the therapist, to help the navigation within the platform, set goals, identify barriers, and answer any questions	Completion of sessions 2 and 3	Completion of sessions 4 and 5, based upon completion of the previous ones	Completion of sessions 6 and 7, based upon completion of the previous ones	Completion of session 8, based upon completion of the previous ones	Completion of session 9, based upon completion of the previous ones	Skype session to set future goals and evaluate the treatment	Participants receive an e-mail linked to a post-treatment questionnaire
Arrange the first Skype session	Participants gain access to the online program (username, password, hyperlink)	Participants access the next sessions if they completed the previous ones	Direct messaging with the therapist	Direct messaging with the therapist	Direct messaging with the therapist	Direct messaging with the therapist	Participants receive an e-mail linked to a post-treatment questionnaire	–
–	–	Direct messaging with the therapist	–	–	–	Arrange the final Skype session	–	–

Rows follow the order of actions undertaken per week.

At the start of each session, participants provided ratings of their developing skills in the categories of openness, awareness, and engagement, on a scale from 0 (never) to 6 (always) in reference to the past three days. These ratings were seen by the therapist, who could then use the information to tailor feedback. During each session, participants were asked about their experience with the material and received individual written feedback from the therapist within 24 hours through secure in-site messaging. The feedback was meant to be individualized, to incorporate any particular challenges specific to PDN, to encourage engagement, and to enhance PF. Participants received weekly reminders to complete sessions through messages generated by the website. When a participant expressed that they wished to drop out, the therapist would ask the reason for discontinuation, via in-site messaging, and whether the participant had any suggested refinements for the treatment that would encourage them to complete all the sessions. When sessions were completed, the therapist could see this; however, data on how frequently/for how long participants practiced the exercises between sessions were not collected. Collecting practice time information would be useful in a larger trial. However, therapist messages served to prompt practice and discuss any barriers or challenges around practicing skills between sessions. At the end of the online sessions, there was a final Skype session with the therapist to encourage participants to set long-term goals, discuss treatment, and make suggestions for improvements. Thus, there was a total of 10 treatment sessions (two Skype and eight online sessions).

### Assessment Procedures

During baseline assessment, participants responded to self-report questions about diabetes and neuropathy duration, medication, comorbidities, age, gender, education, occupation, domestic status, and ethnicity. The participant self-report on the DN4i was used as a screen to support the potential diagnosis of PDN. The DN4i is a psychometrically validated tool used to screen for the possible presence of neuropathic pain. It includes seven interview questions, and a positive screen is indicated by the score of ≥3. The questions include a) pain characteristics (e.g., burning, electric shocks) and b) associated symptoms (e.g., tingling, numbness) [25]. This measure demonstrated good internal consistency in the current sample (Cronbach’s α = 0.72). For participants recruited from the NHS, there was also physician diagnosis of diabetes and PDN.

### Primary Feasibility Outcomes

The primary feasibility outcomes for this study included recruitment, retention, treatment completion rates, and data completeness. Feasibility thresholds for these were defined a priori. The targeted sample to recruit was 30 participants. The aim was to achieve a treatment completion rate of 70% and a follow-up questionnaire completion rate of 80% [15]. Online treatment completion was calculated as the proportion of participants who completed the treatment, defined beforehand, based on Scott’s et al. feasibility trial, as participants completing at least seven out of 10 sessions [15]. Thus, recruitment of 30 participants and achieving 80% follow-up questionnaire completion and 70% treatment completion would support the feasibility of a fully powered RCT.

### Secondary Outcomes

Secondary to the primary feasibility aims outlined above, this study aimed to produce estimates of the magnitude of treatment effect on standard pain outcomes and PF treatment processes as preliminary assessment of potential efficacy. All clinical outcomes were assessed with psychometrically validated and reliable instruments.

#### Standard Pain Outcomes

Pain Intensity and Pain Distress: Pain Scale. Participants rated their average overall pain intensity and distress now and during the past week on a 0 (no pain/distress) to 10 (worst possible pain/distress) numerical scale [26]. This measure has been validated in people with general chronic pain [27].

Depression Symptoms: Patient Health Questionnaire (PHQ-9). The PHQ-9 is a widely used measure of depression symptoms. It is a nine-item questionnaire rated on a 0–3 numerical scale, with the last item rated from “not difficult at all” to “extremely difficult.” A higher score for the sum of the nine items indicates higher levels of depression severity [28]. This measure demonstrated good internal consistency (Cronbach’s α = 0.88) in the current sample.

Functional Impairment: Work and Social Adjustment Scale (WSAS). The WSAS is a five-item questionnaire assessing functional impairment related to one’s health condition. It has been previously used in chronic pain trials [15] and focuses on domains of functioning such as work and hobbies that might be targeted within the treatment. Each item is rated on a 0 (no impairment) to 8 (very severe impairment) scale [29]. This measure demonstrated good internal consistency (Cronbach’s α = 0.94).

Patients’ Global Impression of Change (PGIC). The PGIC is a single-item scale assessing participants overall perception of change after treatment [30]. On this scale, participants report their change as very much improved, much improved, minimally improved, no change, minimally worse, much worse, or very much worse. It is routinely used in trials for chronic pain [31].

#### Theoretically Relevant Treatment Process Variables

Chronic Pain Acceptance: Chronic Pain Acceptance Questionnaire (CPAQ-8). The CPAQ-8 is a reliable measure of chronic pain acceptance, with each item scored on a seven-point scale. The measure reflects pain willingness and activity engagement in the context of pain [32,33]. This measure demonstrated good internal consistency in the current sample (Cronbach’s α = 0.81).

Cognitive Fusion: Cognitive Fusion Questionnaire (CFQ-7). The CFQ-7 is a measure of cognitive fusion or defusion, with items rated on a seven-point scale [34]. Cognitive defusion is the capacity to experience thoughts as just thoughts and not as events as they are directly experienced. This measure demonstrated good internal consistency (Cronbach’s α = 0.97).

Committed Action: Committed Action Questionnaire (CAQ-8). The CAQ-8 is a measure of committed action as defined in the PF model [35]. Its items are rated on a 0 (never true) to 6 (always true) scale, and they reflect the level of flexible commitment in the pursuit of meaningful goals and plans. This measure demonstrated good internal consistency (Cronbach’s α = 0.86).

Self-as-Context: Self-Experiences Questionnaire (SEQ). The SEQ, assesses self-related processes in the PF model, mostly including the capacity to see oneself as distinct from one’s thoughts and feelings. The SEQ is a 15-item questionnaire in which items are rated on a 0 (never true) to 6 (always true) numerical scale [36]. This measure demonstrated good internal consistency (Cronbach’s α = 0.95).

### Statistical Analyses

Data were analyzed with the Statistical Package for Social Science for Windows (version 18.0 IBM; SPSS, Chicago, IL USA). Descriptive statistics, including means and SDs for continuous variables and frequencies and percentages for categorical variables, were calculated for participant characteristics and primary feasibility outcomes.

For the clinical outcome and process variables, including pain distress and pain intensity (pain scale), depression symptoms (PHQ-9), functional impairment (WSAS), chronic pain acceptance (CPAQ-8), cognitive fusion (CFQ-7), committed action (CAQ-8), and self-as-context (SEQ), *t* tests were conducted to determine whether there were differences on the baseline scores for these variables between completers and noncompleters of the treatment. The secondary aim of the study was addressed via effect size calculations and paired *t* test analyses to examine the magnitude of the effect over time on these measures within the single group receiving treatment. In exploratory analyses, mixed between-groups and repeated-measures analysis of variance were used to examine whether treatment completion status was associated with any effects on the measures. The final set of frequency analyses addressed descriptively the participant’s perception of treatment change (PGIC).

Clinically meaningful changes were also calculated following the IMMPACT recommendations, which include the convention of applying a threshold of one-half SD [37]. The value for one-half SD was calculated for each outcome for the whole sample at baseline (pretreatment). A clinically significant effect was identified where the change observed for a participant, in a specific outcome, exceeded one-half SD between pre- and post-treatment.

## Results

### Sample Characteristics

The mean age of participants (SD) was 51.23 (13.30) years. Men represented 56.7% of the sample, and the sample was predominantly white (67%). Equal proportions of the sample either had full-time employment (30%) or were unemployed due to pain (30%), while about a quarter were retired (23.3%). The median DN4i score of all participants was 4.00, and all participants scored higher than the cutoff (an overall score of at least 3) for neuropathic pain. The mean duration of PDN (SD) was 6.97 (1.04) years. Please see Tables 3 and 4 for detailed demographic and clinical characteristics.

**Table 3:** Sample demographic characteristics (N = 30)

	N (%) or M ± SD or Median (Range)
Age, y	51.23 ± 13.30
Age range 21–50 y	15 (50)
Age range 51–80 y	15 (50)
Education, y	15.20 ± 4.92
Gender	
Male	17 (56.7)
Female	13 (43.3)
Ethnicity	
White	26 (86.6)
Asian	2 (6.7)
Mixed	2 (6.7)
Living status	
Alone	5 (16.7)
With partner	10 (33.3)
With child/children	2 (6.7)
With partner and child/children	8 (26.7)
With other relatives	3 (10)
With friends/flatmates	2 (6.6)
Employment status	
Employed full-time	9 (30)
Employed part-time	3 (10)
Unemployed—due to pain	9 (30)
Unemployed—unrelated to pain	1 (3.3)
Student/training—full-time	1 (3.3)
Retired	7 (23.3)
Diagnosis of type 1 diabetes	12 (40)
Diagnosis of type 2 diabetes	18 (60)
DN4i	3.5 (0.00–7.00)
≥4	30 (100)

Range reveals the lowest and highest values, respectively.

DN4i = Douleur Neuropathique 4 interview.

**Table 4:** Diabetes and pain characteristics (N = 30)

	N (%) or M ± SD
Diabetes diagnosis, y	15.50 ± 2.39
Painful diabetic neuropathy duration, y	6.97 ± 1.04
Analgesic medication	
Nonsteroidal anti-inflammatory drugs	4 (13.3)
Anticonvulsants	3 (10.0)
Antidepressants	14 (46.7)
Anti-epileptics	7 (23.3)
Opioids	8 (26.7)
Other	6 (20.0)
No analgesic drugs	5 (16.7)
Comorbidities	
Retinopathy/vision impairment	11 (36.7)
Cardiac infarction	2 (6.7)
Angina pectoris	1 (3.3)
Coronary stent	2 (6.7)
Coronary bypass	2 (6.7)
Diabetic nephropathy	13 (43.3)
Dialysis	1 (3.3)
Leg/foot ulcer	3 (10.0)
Operation on legs	3 (10.0)
Amputation	1 (3.3)
Sleeping disorders	13 (43.3)
Micturition and defecation disorder	2 (6.7)
No comorbidity	7 (23.3)

Note: N is the number of participants, % is the percentage the number of participants represents in the sample, M is the mean and SD is the standard deviation.

### Primary Feasibility Outcomes

In total, 225 people were referred or expressed initial interest in the study, and 30 of these consented to participate (24.6% recruitment) during a three-month recruitment period. One hundred twenty-two (54%) declined to participate, and 73 (32%) were not eligible. Participants were recruited from Guy’s and St Thomas NHS Foundation Trust (N = 2) and online advertisements (N = 28) between October 2018 and December 2018. Twelve (40%) participants completed the online treatment sessions as per the specified completion definition. All participants were retained in the trial (100%), in the sense that they completed all measures, and data completeness was 100%. Reasons for discontinuing treatment can be found in Figure 1. For the 18 people who did not complete treatment, the most frequent reasons were no time (44.4%, N = 8), other health problems (22.2%, N = 4), computer problems (10.5%, N = 2), or other (22.9%, N = 4).

Analyses of pretreatment data for pain intensity and pain distress variables revealed no significant differences between treatment completers and noncompleters. However, comparison of pretreatment scores for depression symptoms, functional impairment, chronic pain acceptance, cognitive fusion, committed action, and self-as-context variables showed large differences between completers and noncompleters. It is notable that, at pretreatment, treatment completers demonstrated lower cognitive fusion and functional impairment and higher levels of committed action, self-as-context, and acceptance than noncompleters. Please see Table 5 for more details.

**Table 5:** Baseline scores on study variables for treatment completers and noncompleters

	Completer	N	Mean	SD	*t*	*D*	*P* Value
Pain intensity (rating scales)	Yes	12	6.50	1.58	0.497	0.19	0.623
	No	18	6.13	2.15			
Pain distress (rating scales)	Yes	12	6.16	2.50	−0.334	−0.13	0.741
	No	18	6.47	2.43			
Depression symptoms (PHQ-9)	Yes	12	11.16	7.28	−2.341	−0.87	0.027
	No	18	17.00	6.27			
Functional impairment (WSAS)	Yes	12	15.92	12.29	−2.033	−0.76	0.052
	No	18	25.44	12.76			
Cognitive fusion (CFQ-7)	Yes	12	11.83	9.31	−2.133	−0.80	0.042
	No	18	21.17	13.08			
Committed action (CAQ-8)	Yes	12	33.17	8.48	2.368	0.88	0.025
	No	18	25.17	9.42			
Self-as-context (SEQ)	Yes	12	64.33	16.77	1.942	0.72	0.062
	No	18	52.28	16.58			
Chronic pain acceptance (CPAQ-8)	Yes	12	26.08	5.40	2.973	1.11	0.006
	No	18	19.61	6.11			

On pain intensity, pain distress, and depression symptom variables, a higher score means worse well-being/functioning, whereas higher scores on process variables (except cognitive fusion measure) indicate higher psychological flexibility.

CAQ-8 = Committed Action Questionnaire; CFQ-7 = Cognitive Fusion Questionnaire; CPAQ-8 = Chronic Pain Acceptance Questionnaire; PHQ-9 = Patient Health Questionnaire; SEQ = Self-Experiences Questionnaire; WSAS = Work and Social Adjustment Scale.

### Secondary Feasibility Outcomes: Clinical Outcomes

At post-treatment, all 18 treatment noncompleters (60% of the overall sample) reported “no change” in their health and functioning compared with before treatment. Among treatment completers (N = 12), all reported that they felt “improved” (N = 10) or “very much improved” (N = 2).

Each of the variables from the clinical outcome and process measures was examined for normality using histograms, Q-Q plots, skewness, and kurtosis. None of these showed significantly skewed distributions or outliers expected to adversely affect the analyses. See Table 5 for group means and standard deviations on study variables.

Effect size calculations and paired *t* test analyses of pre- and post-treatment scores for the full sample revealed small effects over time for depression symptoms and functional impairment and medium effects for pain intensity and pain distress, chronic pain acceptance, cognitive fusion, committed action, and self-as-context. These results included a mix of improvements in some variables and deterioration in others, owing particularly to deterioration in the larger treatment noncompleters (Table 6). However, the majority of the sample did not complete treatment, and therefore, an improvement in the full sample analysis was not necessarily expected. The analysis of time by completer, which was conducted, showed that some of these variables improved among the completers.

**Table 6:** Paired *t* test uncontrolled analysis and repeated-measures ANOVA examining psychological flexibility variables in completers (N = 12) and noncompleters (N = 18)

Paired *T* Test Uncontrolled Analysis									
	Pretreatment Scores			Post-treatment Scores					
	Mean		SD	Mean		SD	*t*	*D*	*P* Value
Pain intensity	6.28		1.92	5.05		3.64	1.59	0.42	0.124
Pain distress	6.35		2.42	5.28		3.88	1.29	0.32	0.208
Depression symptoms	14.67		7.187	14.27		9.00	0.26	0.05	0.795
Functional impairment	21.64		13.24	22.67		15.91	−0.34	−0.07	0.736
Cognitive fusion	17.43		12.44	24.87		15.15	−2.58	−0.53	0.015
Committed action	28.37		9.76	22.50		15.71	2.23	0.44	0.034
Self-as-context	57.10		17.43	39.53		29.37	3.41	0.72	0.002
Chronic pain acceptance	22.20		6.58	24.00		4.15	−1.74	−0.32	0.092

Time*completer interaction effects									

		Pretreatment Scores		Post-treatment Scores					
							
	Completer	Mean	SD	Mean	SD	MS	*F*	*d_ppc2_* *	*P* Value

Pain intensity	Yes	6.50	1.58	0.83	0.72	196.54	82.89	3.76	0.000
	No	6.13	2.15	7.86	1.17				
Pain distress	Yes	6.16	2.50	0.75	0.45	189.23	48.29	2.93	0.000
	No	6.47	2.43	8.31	1.19				
Depression symptoms	Yes	11.16	7.28	4.08	3.23	446.67	21.94	−1.65	0.000
	No	17.00	6.27	21.06	3.06				
Functional impairment	Yes	15.92	12.29	3.92	3.32	1,698.68	20.55	−1.71	0.000
	No	25.44	12.76	35.17	3.33				
Cognitive fusion	Yes	11.83	9.31	7.17	2.62	1,464.10	19.19	−1.08	0.000
	No	21.17	13.08	36.67	4.33				
Committed action	Yes	33.17	8.48	40.25	5.15	1,677.03	35.26	2.36	0.000
	No	25.17	9.42	10.67	5.78				
Self-as-context	Yes	64.33	16.77	73.42	7.90	7,102.23	44.44	2.64	0.000
	No	52.28	16.58	16.94	8.98				
Chronic pain acceptance	Yes	26.08	5.40	28.33	2.27	2.03	0.12	−0.13	0.729
	No	19.61	6.11	21.11	2.00				

ANOVA = analysis of variance.

* *d_ppc2_* (pretest–post-test–control): according to Morris [38].

### Exploratory Analyses of Treatment Completion and Clinical Outcomes

Large interaction effects between time point and treatment completion were observed across all variables examined, except for chronic pain acceptance, where the effect was very small. The large effects included pain intensity, pain distress, depression symptoms, functional impairment, cognitive fusion, committed action, and self-as-context. Please see Table 6 for more details.

This was confirmed when data were split into completers and noncompleters of the treatment. For completers, there were significant improvements within-group over time, including a large effect for pain intensity and pain distress, depression symptoms, and functional impairment. These results appear superior to those of participants who did not complete the treatment, who generally deteriorated.

Over time, completers improved and demonstrated a large effect for committed action compared with noncompleters, who had lower scores and a similarly large effect in the opposite direction. Completers showed a medium effect for cognitive fusion and self-as-context. On the other hand, noncompleters over time reported significantly higher levels of cognitive fusion and lower levels of self-as-context. There were medium effects for completers and noncompleters for chronic pain acceptance.

### Clinically Meaningful Changes

The percentage of completers and noncompleters who experienced clinically meaningful changes can be found in Table 7. At post-treatment, the majority of treatment completers showed meaningful improvements for seven out of eight of the outcome variables. The exception was chronic pain acceptance, where 41.7% meaningfully improved, while 50% did not meaningfully change. Very few of the completers deteriorated meaningfully. For four of the outcomes, there were none, and for the others, there was one participant. For the noncompleters, the picture of meaningful change was more mixed. In six of eight outcomes, 72% or more of the participants either showed no meaningful change or meaningfully deteriorated. For just two outcomes, the majority meaningfully improved (for committed action and self-as-context, which was unexpected). For pain intensity, pain distress, and depression, the majority of noncompleters deteriorated.

**Table 7:** Percentages of completers and noncompleters who made clinically meaningful improvements, showed no change, and deteriorated post-treatment

	Completers (N = 12)			Noncompleters (N = 18)		
	Improved (%)	No Change (%)	Deteriorated (%)	Improved (%)	No Change (%)	Deteriorated (%)
Pain intensity	12 (100.0)	0 (0.0)	0 (0.0)	2 (11.1)	4 (22.2)	12 (66.7)
Pain distress	12 (100.0)	0 (0.0)	0 (0.0)	3 (16.7)	4 (22.2)	11 (61.1)
Depressive symptoms	8 (66.7)	4 (33.3)	0 (0.0)	2 (11.1)	6 (33.3)	10 (55.6)
Functional impairment	7 (58.3)	5 (41.7)	0 (0.0)	1 (5.6)	10 (55.6)	7 (38.9)
Cognitive fusion	5 (41.7)	6 (50.0)	1 (8.3)	1 (5.6)	4 (22.2)	13 (72.2)
Committed action	8 (66.7)	3 (25.0)	1 (8.3)	16 (88.9)	0 (0.0)	2 (11.1)
Self-as-context	7 (58.3)	4 (33.3)	1 (8.3)	18 (100.0)	0 (0.0)	0 (0.0)
Chronic pain acceptance	5 (41.7)	6 (50.0)	1 (8.3)	5 (27.8)	7 (38.9)	6 (33.3)

Percentages are rounded up to 1 decimal digit.

## Conclusions

The aim of this study was to assess the feasibility of conducting a larger RCT of online ACT for people with PDN. The targeted sample size was recruited (N = 30), and all participants were retained in the trial and completed follow-up questionnaires. However, the treatment completion rate was 40%, which was below the prespecified feasibility target of 70%. Hence, partial feasibility was found for the research and treatment methods for evaluating online ACT for PDN in a larger RCT. The treatment completion rate here is considered inadequate to justify proceeding to a full-scale trial until some modifications to enhance treatment engagement are designed and demonstrated.

The treatment completion rate for the current treatment was 40%, which is lower than a Dutch trial (72%) [19], a German trial (60%) [39], and a UK trial (61%) [15] of online ACT for general chronic pain. In the current study, there were differences at baseline between treatment completers and noncompleters, even though the sample was largely self-selected online, and these differences may underlie the high dropout rate. Particularly, noncompleters had relatively higher levels of cognitive fusion, depressive symptoms, functional impairment, and lower levels of committed action, pain acceptance, and self-as-context. This appears not to have been found in other similar studies [15,19,39] and may be unique to the PDN population, perhaps due to the complexity or nature of neuropathic pain, or it could be due to some unique aspect of the setting or methods used here. As this is a one-time finding in a small sample, it is too soon to determine what it means.

If further research again shows that factors such as higher levels of cognitive fusion, depressive symptoms, and functional impairment and lower levels of committed action, pain acceptance, and self-as-context are associated with inadequate treatment completion, then it could be used either in selectively allocating participants to treatment, as targets for pretreatment intervention, or as a basis for redesign of the treatment methods or content. Presumably, selecting participants with relatively lower depression or functioning impairment as a group may result in better completion rates.

It may be that participants with particularly low levels of PF or severe depression and high pain interference require more intensive psychological therapy, such as that delivered in a face-to-face setting (individual or group). However, it is known from previous studies that online treatment completion rates can be low, apparently due to problems with the use of technology, barriers due to poor health, or low motivation [40]. Based on our data, it is not known whether it was the ACT approach itself, aspects of online delivery, requirements inherent in any psychological treatment, or all of the above that was unacceptable to participants. Most of those who did not complete treatment reported a lack of time. Another possible explanation represented in supplemental background information was that 11/30 reported some degree of visual impairment, which would make it difficult for them to complete treatment that mainly consisted of videos. Each of these possibilities deserves further consideration.

Future research may explore treatment engagement through a qualitative study to investigate PDN participants’ preferences for delivery format and views about ACT as a treatment approach. The model underlying ACT suggests that a core set of behavioral processes underlie the treatment impact and that a standard package of this treatment ought to be generally applicable. However, our data suggest that the treatment may need to be better tailored in a PDN context. This could be achieved by providing specific case examples of PDN throughout and orienting participants to problem areas specific to PDN such as fear of falling [2,3]. Treatment might also focus more explicitly on improving sleep. Given that 13/30 of the participants reported significant sleeping problems, this could be a motivating element if added to the treatment. A qualitative study could help to further identify specific problem areas within PDN for which to apply ACT skills. This could contribute to better tailoring the treatment for this population and enhance engagement.

Another way to potentially enhance treatment engagement is to allow the treatment to be more dynamically customizable around each individual. This could include remotely assessing each case intensively over time, supporting the selection of treatment modules that are personalized, and delivering only the modules particular participants need and not the ones they do not, leading to more rapid and efficient benefits from treatment [41]. In theory, a customized modular treatment guided by daily data gathering could pick up on, and intervene in, engagement lapses to promote better completion rates. The treatment components delivered here could certainly be repackaged to operate in this fashion.

The observed uncontrolled effect sizes on the clinical outcomes and process measures ranged from small to large at three months, favoring a decrease of depression symptoms, functional impairment, pain intensity, and pain distress and an increase of chronic pain acceptance and committed action in treatment completers. Although clinical outcome results are highly preliminary, the large reduction in pain differs from other ACT trial results. This may be relevant to the observation in a recent cross-sectional survey that PF may play a smaller role, compared with pain intensity, in relation to distress and disability in the PDN population [13].

The rate of clinically meaningful results for treatment completers across outcomes are encouraging. At post-treatment, all treatment completers showed meaningful improvement in at least three variables, 83.3% in at least four variables, 41.7% in at least five, 33.3% in at least six, 25% in at least seven, and 16.7% in eight. On the other hand, all noncompleters showed a meaningful deterioration in at least two variables, and half of noncompleters deteriorated in at least half (four of eight) of the outcomes. These results may provide “proof of concept” that ACT can benefit people suffering from the effects of PDN, provided that they can be supported to complete the treatment sessions. On the other hand, support for applying ACT in this context may only apply to people who are relatively higher in functioning and PF.

A notable result is the number of clinical outcome and process measures on which those who did not complete treatment worsened during the three-month interval examined. In fact, on every measure, with the exception of pain acceptance, the noncompleters were worse at the end of the trial compared with the beginning. In several cases, these declines were significant and large, and in all cases this was unexpected. This perhaps reflects natural variability in PDN, and perhaps this contributed in some way to noncompletion, but this is only speculation [40,42]. Another possible explanation might be that the treatment did not adequately target key areas of need for participants. For example, depression is highly prevalent in people with diabetes and in those with diabetes complications. Therefore, not only does this population have significant levels of pain, but they also have comorbid disability because of PDN, like balance and mobility problems, and associated microvascular comorbidities, such as retinopathy and nephropathy [43]. These comorbidities were not adequately measured or reported for this sample. Qualitative interviews with the noncompleters would have allowed us to determine the reason for these changes and the main reason for discontinuing treatment. Also, a revised version of treatment might help participants to practice applying these skills more broadly to other diabetes-related problems, which might be considered to have a larger impact on their functioning and quality of life.

Possibly, noncompleters were experiencing symptoms of PDN during their engagement in this treatment, became more conscious of their experienced difficulties, and were willing to report them. Additionally, it is possible that the nature of neuropathic pain is responsible for noncompletion of treatment. It may be relevant that neuropathic pain is different pathophysiologically, compared with other chronic pain conditions, with the dominant component of neuroplastic changes within the nervous system [44]. These speculations deserve study.

In this study, the most commonly suggested refinements by the noncompleters, coming from comments in the experiential exercises or the last Skype session with the therapist, were to shorten audios and videos, add more face-to-face sessions, provide more educational material on diabetes and neuropathy, and provide additional printed materials to supplement the online content. We note, however, that the total time for all online content was just 150 minutes, or an average of less than 19 minutes for each online session. Nonetheless, it could be possible to provide choices around longer exercises by more clearly alerting participants regarding the length and providing them with scheduling options (now or later when there is more time available) or by providing a choice for several shorter exercises in the place of a lengthy one. A missed opportunity here, to investigate treatment noncompletion, would have been to include in-depth exit interviews with participants who dropped out. Unfortunately, this method was not possible in the current study due to lack of resources. Such a study could provide more detailed feedback on reasons for dropping out or losing interest.

This study has several limitations. First, the study design does not allow for causal interpretations for observed changes in outcomes, as this was not an RCT. Second, even though the recruitment target was reached (N = 30), this is a small sample with high dropout rates (60%), which may lead to limited reliability and precision of our estimates and limited power for all of the mean comparisons. The sample was also too small to conduct meaningful analyses to identify characteristics associated with a favorable response to treatment responses. Third, the fact that participants were self-selected to take part in the treatment means that results may not generalize to the wider population of people with PDN in need of treatment. Also, as the majority of participants were recruited from online portals, and even though we used the DN4i and self-reported questions for diabetes and PDN diagnosis, there is the possibility that participants did not fulfil more stringent diagnostic criteria for PDN. It is worth noting that the treatment applied here was designed for people with chronic pain in general. In retrospect, this is possibly not an ideal test for the specific feasibility for people with PDN, and a more tailored version of treatment may ultimately be more feasible. Finally, it is recognized that a different sample and longer follow-up may yield different results. The generalizability and reliability of the results still need to be established.

Despite these limitations, this is the first feasibility study of online ACT in people with PDN. Based on low completion rates, a larger RCT testing efficacy is not feasible for the current online ACT treatment as examined here. Future research is encouraged to specifically address the problem of low treatment completion, possibly including active patient involvement and qualitative work. Further tailoring of research methods and treatment to specifically fit PDN may be needed. Another avenue, at the same time, is simply greater individualization. This could include identifying the defining features of individuals who will both engage in and achieve clinically meaningful benefits from the treatment model here, and those who will not. It could also include making this treatment more sensitive to whoever encounters it by breaking it into modules and personalizing the delivery of these based on assessment data.

## Authors’ Contributions

KK, the first author, responsible for the work as a whole, recruited patients, delivered the treatment, collected and analyzed the data, and produced the first draft of the manuscript. LM and KW contributed to the conception, design of the study, and research plan, offered their guidance on protocol and selection of assessment tools, and edited and approved the final version of the manuscript. WS contributed by offering guidance throughout the treatment delivery and statistical analysis plan and revised, edited, and approved the manuscript. EG supported the project, offered guidance and expertise, and revised, edited, and approved the final version of the manuscript.

## Notes

Funding sources: This study was funded via a PhD fellowship grant from Diabetes UK (Ref: 16/0005540). Also, this research is independent work partly supported by the National Institute for Health Research (NIHR Postdoctoral Fellowship, Dr. Whitney Scott, PDF-2015-08-059).

Disclaimer: The views expressed in this publication are those of the authors and not necessarily those of the National Institute for Health Research or Diabetes UK.
